# DMXAA Causes Tumor Site-Specific Vascular Disruption in Murine Non-Small Cell Lung Cancer, and like the Endogenous Non-Canonical Cyclic Dinucleotide STING Agonist, 2′3′-cGAMP, Induces M2 Macrophage Repolarization

**DOI:** 10.1371/journal.pone.0099988

**Published:** 2014-06-18

**Authors:** Charlene M. Downey, Mehrnoosh Aghaei, Reto A. Schwendener, Frank R. Jirik

**Affiliations:** 1 Department of Biochemistry and Molecular Biology, University of Calgary, Calgary, Alberta, Canada; 2 The McCaig Institute for Bone and Joint Health, University of Calgary, Calgary, Alberta, Canada; 3 Institute of Molecular Cancer Research, Laboratory of Liposome Research, University of Zurich, Zurich, Switzerland; University of Sheffield, United Kingdom

## Abstract

The vascular disrupting agent 5,6-dimethylxanthenone-4-acetic acid (DMXAA), a murine agonist of the stimulator of interferon genes (STING), appears to target the tumor vasculature primarily as a result of stimulating pro-inflammatory cytokine production from tumor-associated macrophages (TAMs). Since there were relatively few reports of DMXAA effects in genetically-engineered mutant mice (GEMM), and models of non-small cell lung cancer (NSCLC) in particular, we examined both the effectiveness and macrophage dependence of DMXAA in various NSCLC models. The DMXAA responses of primary adenocarcinomas in *K-ras^LA1/+^* transgenic mice, as well as syngeneic subcutaneous and metastatic tumors, generated by a *p53^R172HΔg/+^; K-ras^LA1/+^* NSCLC line (344SQ-ELuc), were assessed both by *in vivo* bioluminescence imaging as well as by histopathology. Macrophage-dependence of DMXAA effects was explored by clodronate liposome-mediated TAM depletion. Furthermore, a comparison of the vascular structure between subcutaneous tumors and metastases was carried out using micro-computed tomography (micro-CT). Interestingly, in contrast to the characteristic hemorrhagic necrosis produced by DMXAA in 344SQ-ELuc subcutaneous tumors, this agent failed to cause hemorrhagic necrosis of either 344SQ-ELuc-derived metastases or autochthonous *K-ras^LA1/+^* NSCLCs. In addition, we found that clodronate liposome-mediated depletion of TAMs in 344SQ-ELuc subcutaneous tumors led to non-hemorrhagic necrosis due to tumor feeding-vessel occlusion. Since NSCLC were comprised exclusively of TAMs with anti-inflammatory M2-like phenotype, the ability of DMXAA to re-educate M2-polarized macrophages was examined. Using various macrophage phenotypic markers, we found that the STING agonists, DMXAA and the non-canonical endogenous cyclic dinucleotide, 2′3′-cGAMP, were both capable of re-educating M2 cells towards an M1 phenotype. Our findings demonstrate that the choice of preclinical model and the anatomical site of a tumor can determine the vascular disrupting effectiveness of DMXAA, and they also support the idea of STING agonists having therapeutic utility as TAM repolarizing agents.

## Introduction

Strategies targeting the tumor vasculature represent an attractive approach in cancer therapy, and as such there has been much interest in a class of drugs known as vascular disrupting agents (VDA) [1], [2]. The VDA, 5,6-dimethylxanthenone-4-acetic acid (DMXAA; a.k.a. ASA-404) specifically targets immature and unstable vasculature of solid tumors, leading to thrombosis, hemorrhage, and necrosis [3]. In a host of preclinical studies involving many different tumor types and primarily in subcutaneous tumor models, DMXAA has demonstrated potent anti-tumor activity [4]–[6]. In addition, synergies were observed when DMXAA was used in conjunction with cytotoxic chemotherapeutic agents that targeted the viable rim of tumor cells that typically survive DMXAA treatment [3], [7]–[9].

There is evidence that the actions of DMXAA on tumor vasculature involve both direct and indirect effects, via targeting of the endothelium, and macrophages, respectively. The latter appear to be the most important, and are the result of DMXAA-triggered release of tumor-associated macrophage (TAM)-derived factors, such as TNF-α and NO [5], [7], [10]–[12], together with contributions from various other cytokines and chemokines [2], [6]–[8]. Following success in preclinical studies, the impetus for moving DMXAA into a Phase III trial for NSCLC stemmed largely from the observed increase in overall survival reported in a previous Phase II trial [13], [14]. However, the larger trial, and other Phase II trials, failed to produce favorable outcomes [15]–[17]. This raised the question as to why there was such a discrepancy between the positive results obtained using pre-clinical animal models and the results in the clinic. Recently, it was shown that DMXAA exhibits differential effects on murine and human macrophages [18], and also that the stimulator of interferon genes (STING), was a receptor for DMXAA [19]–[21]. The finding that DMXAA was unable to activate human STING provided a salient explanation for the failure of this agent in the human clinical trials [20], [21].

With the goal of gaining further insight into additional variables accounting for the differential effects of DMXAA between pre-clinical and clinical trials, we examined the effects of this agent in several mouse models, including: (*a*) syngeneic subcutaneous and metastatic tumors due to a cell line (344SQ-ELuc) derived from the *p53^R172HΔg/+^ K-ras^LA1/+^* genetically engineered mutant mouse (GEMM) model of NSCLC [22]–[24]; (*b*) primary lung adenocarcinomas arising in the *K-ras^LA1/+^* model of NSCLC; and (*c*) subcutaneous and metastatic tumors due to the human MDA-MB-231 breast cancer cell line [25]. Consistent with previous pre-clinical studies, DMXAA led to massive hemorrhagic necrosis in subcutaneously grown breast cancer and NSCLC cell line tumors. In contrast, neither autochthonic lung adenocarcinomas arising in *K-ras^LA1/+^* transgenic mice [26], nor metastases derived from intracardiac injections of syngeneic 344SQ-ELuc NSCLC cells showed responses to DMXAA administration. In addition, we found that clodronate liposome-mediated macrophage depletion [27] abrogated DMXAA-induced intra-tumoral hemorrhagic necrosis in 344SQ-ELuc subcutaneous tumors.

Although the macrophage-derived factors thought to mediate the anti-tumor effects of DMXAA are characteristic of M1 polarized cells, we found that the murine NSCLC TAMs were primarily M2-like. Thus, the responses of M1 and M2 macrophages to DMXAA were investigated. We found that M2 polarized bone marrow-derived macrophages, as well as M2-like TAMs could be re-polarized to an M1 phenotype by DMXAA. We further found that the endogenous STING ligand, 2′3′-cGAMP, produced a similar repolarization phenotype. TAM re-education represented a plausible mechanism whereby M2-like TAMs were able to mediate vascular disruption in response to DMXAA. Our results lend support to the idea of using STING agonists as TAM repolarizing agents, and they also highlight the importance of testing agents on a variety of preclinical models. In addition, our study highlights the growing awareness of the utility of GEMMs for preclinical drug studies [28], [29].

## Results

### NSCLC TAMs and Clodronate Liposome-mediated TAM Depletion

**Figure 1:**
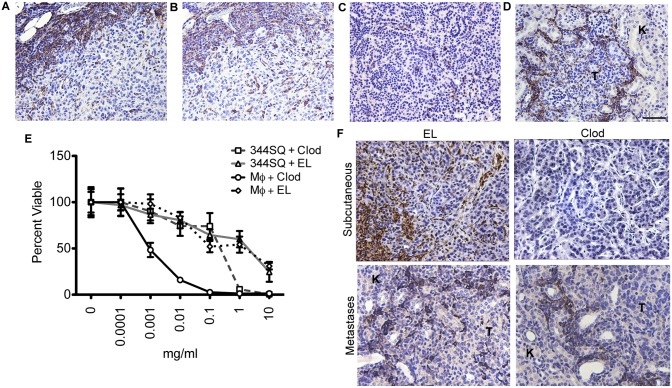
Effectiveness of clodronate-mediated TAM depletion varied depending on tumor site. Representative 344SQ-ELuc subcutaneous tumor sections were stained with antibodies against Iba-1 (**A**) and Arg-1 (**B**), demonstrating abundant M2-like macrophages primarily at the tumor periphery. In contrast, much lower and variable macrophage infiltration was present in either the *K-ras^LA1/+^* primary NSCLCs (**C**) or in 344SQ-ELuc metastases (**D**). (**E**) MTT assay conducted on BMDM (Mφ) or 344SQ-ELuc cells to assess potential cytotoxicity in response to either Clodrolip (Clod) or empty liposomes (EL). (**F**) Representative 344SQ-ELuc subcutaneous tumor and kidney metastases sections from Clod-treated mice were stained with Iba-1 showing that TAM depletion only occurred in subcutaneous tumors (T = tumor, K = kidney). Scale bar = 100 µm (A–B) 50 µm (C, D, F). Data represent the mean ± SEM.

Consistent with the evidence that macrophages play an important role in the action of DMXAA [11], [30], we found that 344SQ-ELuc subcutaneous tumors contained large numbers of infiltrating macrophages, as detected by ionized Ca^2+^-binding adaptor (Iba)-1 immunostaining. These cells were concentrated primarily at the tumor periphery (**Figure 1A**), and the vast majority of the TAMs were positive for the murine M2 marker, arginase (Arg)-1 (**Figure 1B**). In contrast, spontaneously arising *K-ras^LA1/+^* lung adenocarcinomas contained a relative paucity of TAMs, with only a few scattered cells located at the tumor periphery (**Figure 1C**). To obtain metastases, 344SQ-ELuc NSCLC cells were introduced via intracardiac, left-ventricle (LV), injection in syngeneic mice. This route reproducibly resulted in multiple kidney, adrenal gland, lung, and visceral fat pad metastases (**Figure S1**). These metastases contained variable levels of macrophage infiltration (**Figure 1D**) but at considerably lower levels than were observed in the 344SQ-ELuc subcutaneous tumors.

To investigate the role of macrophages in mediating DMXAA vascular disruption, we depleted macrophages using clodronate-encapsulated liposomes (Clodrolip) [27]. A comparison of the effects of *in vitro* Clodrolip treatment of 344SQ-ELuc cells and bone marrow-derived macrophages (BMDM) demonstrated an ∼100-fold greater sensitivity of macrophages (**Figure 1E**). *In vivo*, Clodrolip led to an ∼50% decrease in CD11b^+^ F4/80^+^ marrow cell populations (**Figure S2A–B**), and although we obtained ∼100% depletion of TAMs within subcutaneous 344SQ-ELuc tumors, no evidence of TAM depletion was seen in 344SQ-ELuc metastases (**Figure 1F**). TAMs can play a supportive role during tumor development, and consistent with this, we found that Clodrolip-mediated TAM depletion slowed the growth of 344SQ-ELuc subcutaneous tumors while growth of metastases was unaffected, presumably due to the inability to obtain macrophage depletion (**Figure S2C–F**).

### Depletion of Macrophages in Subcutaneous 344SQ-ELuc Tumors Alters the Response to DMXAA

**Figure 2:**
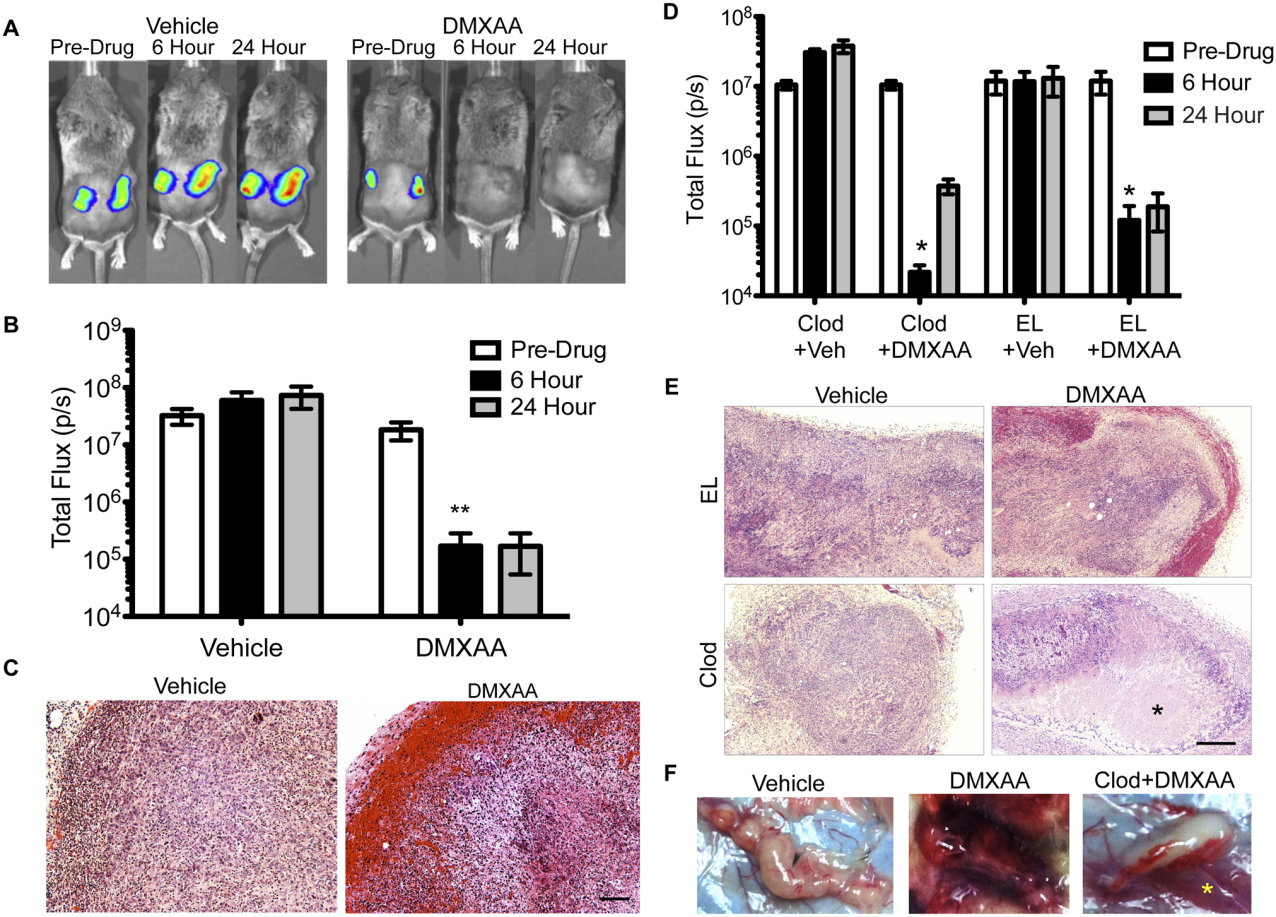
TAM depletion prevented DMXAA-induced hemorrhagic necrosis of subcutaneous NSCLC. (**A**) BLI of bilateral 344SQ-ELuc subcutaneous tumors in a representative syngeneic 129/Sv mouse at day 14 post-cell injection. Mice were randomized into two groups and administered a single i.p. dose of 25 mg/kg DMXAA or DMSO vehicle and imaged again at 6 and 24 hours (N = 10). Regions of interest (ROIs) drawn over tumors or whole body, to quantify photon emission rates, revealed a dramatic loss of signal intensity following DMXAA treatment of mice with subcutaneous tumors. (**B**) (**p≤0.01). (**C**) Histology of representative subcutaneous tumors taken at 24 hours post-DMXAA treatment demonstrated hemorrhagic rims and necrosis (scale bars = 100 µm). (**D**) 344SQ-ELuc subcutaneous tumors were established in Clod or EL treated mice which were then given DMXAA or vehicle control (N = 6 Clod plus DMXAA; N = 3 controls). Quantification of ROI demonstrated a significant drop in photon emission rates in response to DMXAA (*p<0.05). (**E**) Histology of representative tumors at 24 hours demonstrates absence of hemorrhage at the tumor periphery in the Clod plus DMXAA treated mice, however, necrosis still occurred (*) (scale bar = 100 µm). (**F**) Representative gross pathology of subcutaneous 344SQ-ELuc tumors following DMXAA treatment, showing extensive hemorrhage both within and in the immediate vicinity of the tumor. Note that Clod plus DMXAA treated tumors did not exhibit intra-tumoral hemorrhage, but rather showed thrombosis and hemorrhage confined to larger feeding vessels (yellow asterisk). Data represent the mean ± SEM.

Consistent with previous reports [22], 344SQ-ELuc NSCLC subcutaneous tumors respond dramatically to DMXAA, with a marked (∼2-logs) decrease in bioluminescence (BLI) signals post-drug injection (**Figure 2A–B**). This was accompanied by vascular thrombosis and hemorrhage in the tumor periphery, and by the development of extensive central necrosis (**Figure 2C**). The drop in BLI following DMXAA treatment was not due to direct tumor cell toxicity since DMXAA had no detrimental effect on 344SQ-ELuc cell viability (**Figure S3**). Instead, tumor BLI signal loss was attributable to greatly diminished blood, and hence luciferin substrate, perfusion which would diminish ATP-dependent light production. While decreased perfusion could conceivably have resulted from reversible vasoconstriction, given the massive tumor necrosis observed, it was more likely that decreased light emission was the result of tumor vessel thrombosis and rupture.

Since TAMs could be efficiently depleted by Clodrolip in the 344SQ-ELuc subcutaneous tumors, we evaluated the responses of these tumors to DMXAA. Mice treated with empty liposomes (EL) served as the control group. DMXAA still provoked a dramatic drop (∼2-logs) in bioluminescence signal intensity (**Figure 2D**), however, this occurred in the absence of the characteristic intra-tumoral thrombosis and hemorrhage. Instead, DMXAA led to massive non-hemorrhagic, ‘dry’, necrosis (**Figure 2E**). The latter appeared to result from DMXAA-induced occlusion of tumor-feeding vessels (**Figure 2F**). Although it was conceivable that Clodrolip had sensitized the feeding vessels to DMXAA, this result suggested the VDA effects of DMXAA were not confined to the intra-tumoral vasculature, but rather that tumor-feeding arterioles had also been compromised by this agent. Indeed, DMXAA may have a direct effect on endothelial cells [3], [7]–[9]. While it is plausible that clodronate treatment may have sensitized the tumor vasculature, for example, by increasing vessel fragility or interfering with pericyte coverage, we have found that large tumor-feeding vessel thrombosis is also present after DMXAA administration to non-Clodrolip exposed mice (data not shown). The results suggest that DMXAA mediates its effects in two ways: by causing macrophage-induced intra-tumoral microvessel disruption and by causing thrombosis of tumor-feeding vessels. Regardless, it was clear that subcutaneous 344SQ-ELuc tumor TAM depletion was effective in preventing DMXAA-induced intra-tumoral hemorrhagic thrombosis.

### Both DMXAA and the Non-canonical Cyclic Dinucleotide 2′3′-cGAMP are able to Re-educate M2 Macrophages towards an M1 Pro-inflammatory Phenotype

**Figure 3:**
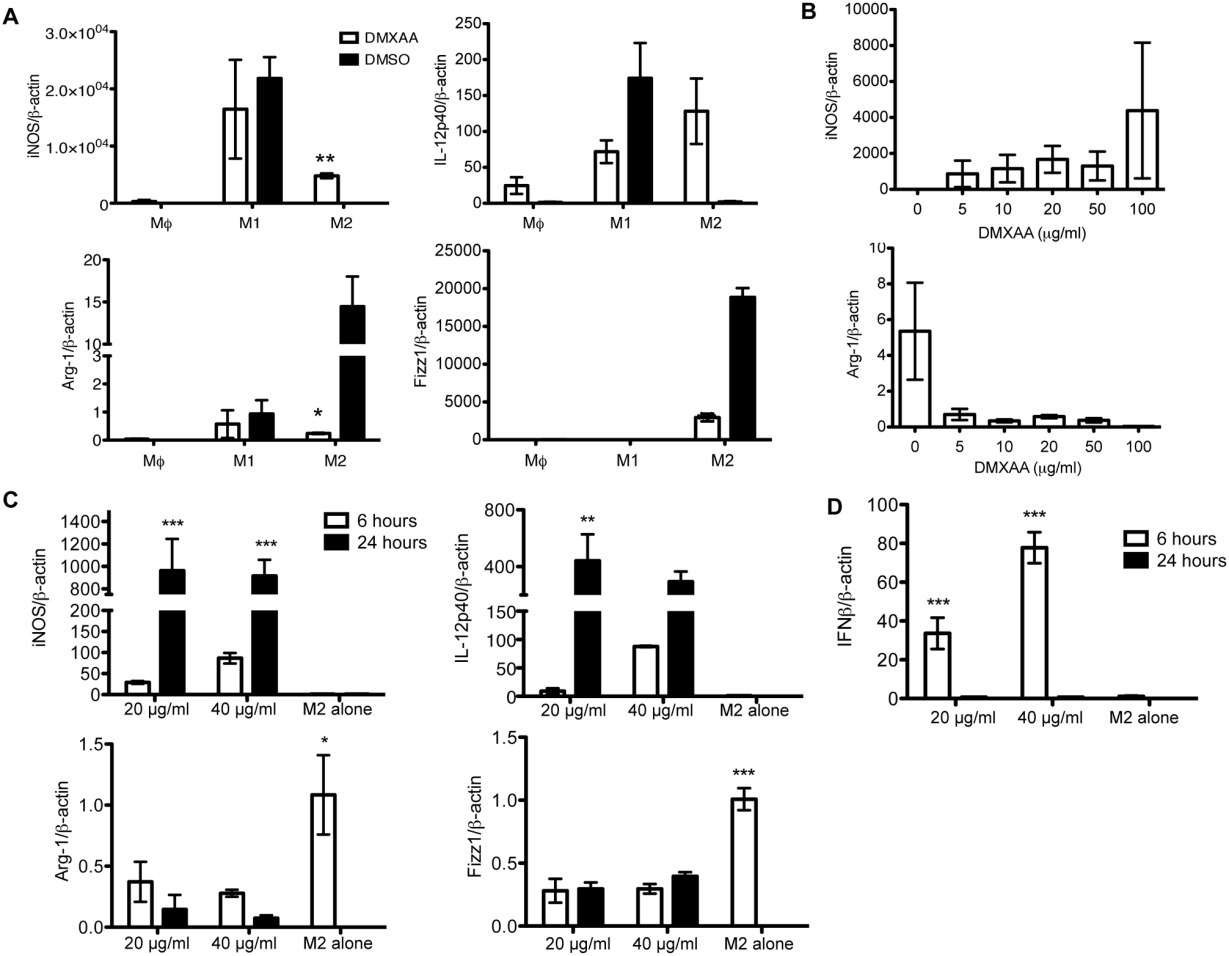
Exposure to either DMXAA or 2′3′-cGAMP repolarized M2 macrophages towards an M1 phenotype *in vitro*. (**A**) Triplicate samples of non-polarized macrophages (Mφ), M1-, and M2-polarized macrophages were exposed to 20 µg/ml DMXAA for 24 hours *in vitro* and RNA transcript levels measured by qRT-PCR. DMXAA down-regulated Arg-1 and Fizz1 expression, while increasing expression of iNOS and IL-12p40. (**B**) Shows reciprocal changes in Arg-1 and iNOS expression in M2 cells in response to increasing concentrations of DMXAA (N = 3). (**C**) RNA transcripts were also taken from triplicate samples of M2-polarized macrophages exposed to 20 or 40 µg/ml 2′3′-cGAMP plus LF2000 for 6 and 24 hours *in vitro*. LF2000 alone served as the control (designated as ‘M2 alone’ in the graphs). 2′3′-cGAMP led to down-regulation of Arg-1 and Fizz1, and dramatic increases in iNOS and IL-12p40 expression in a dose-dependent manner. (**D**) IFN-β induction provided an indication of STING activation in response to 2′3′-cGAMP, with strong inductions at 6 hours that returned to baseline by 24 hours (*p<0.05, **p<0.01, ***p<0.001).

Since the intra-tumoral hemorrhagic response of subcutaneous 344SQ-ELuc tumors to DMXAA was dependent on TAMs, we next examined the effect of DMXAA directly on macrophages. DMXAA is known to stimulate the production of cytokines, such as TNFα, CXCL10 (IP-10), MIP-1α, and MIP-1β, [7], [10], in order to mediate vascular disruption, and these factors are typical of M1 macrophage responses. However, we found that the majority of 344SQELuc TAMs were M2-like (**Figure 1A–B**). Hypothesizing that DMXAA would alter macrophage polarization, supernatant cytokine and chemokine profiles of DMXAA-treated BMDM M1- and M2-polarized macrophages (**Figure S4 A**) were evaluated by a cytokine/chemokine discovery array. The top fold-changed cytokines are listed in **Table 1** (for the raw data see **Table S1**). Although the majority of the up-regulated cytokines were characteristic of M1-polarized cells (e.g. CXCL10, CXCL9, IL-1α and β), we also found that compared to the M1 cells, the IL-4 polarized M2 macrophages displayed 2–10 fold greater inductions of these factors in response to DMXAA, indicative of their re-education towards an M1 phenotype [31]–[33]. Furthermore, analysis of RNA transcripts provided additional evidence that M2 macrophages had been shifted towards an M1 phenotype, as demonstrated by decrease in the M2 markers, Arg-1 and Fizz1, and acquisition of the M1 markers, iNOS and IL-12p40 (**Figure 3A**). The repolarizing effect of DMXAA was even evident at relatively low concentrations (e.g. 5 µg/ml) of this agent (**Figure 3B**). Consistent with STING-TBK1 pathway activation [34], by reverse-phase protein array, we found that DMXAA-mediated upregulation of the NF-κB pathway as shown by increased p65 phosphorylation in M2 macrophages (**Figure S4B**).

**Table 1:** DMXAA-induced factors in M1- and M2-polarized BMDMs.

	M1 + DMXAA	M2 + DMXAA
Factor	(fold change ± SEM)	(fold change ± SEM)
IL-1α	[1.0±0.3]	1.9±02
IL-1β	1.9±0.4	2.8±1.2
IL-6	4.1±0.4	1.2±0.4
IP-10 (CXCL10)	36.5±2.3	357.5±18.4**
MCP-1 (CCL2)	6.9±0.6	9.6±2.2
MIG (CXCL9)	1.1±0.004	43.9±22.7
MIP-1α	17.3±2.7	76.6±19.9
MIP-1β	109.9±14.1	247.8±41.8
RANTES (CCL5)	1.1±0.05	181.8±48.8
TNF-α	2.4±0.3	5.8±1.5
VEGF	[1.8±0.4]	[2.5±0.9]

Note: Brackets indicate a negative fold-change (**p≤0.01).

Since one of the targets of DMXAA is murine STING, we tested another STING agonist, the non-canonical endogenous cyclic dinucleotide 2′3′-cGAMP [35], [36] on M2 macrophages. As was the case with DMXAA, we found that 2′3′-cGAMP administration to M2-polarized macrophages dramatically increased interferon-β, as well as expression of M1 markers iNOS and IL-12p40, and this was accompanied by decreased expression of the M2 markers, Arg-1 and Fizz1 (**Figure 3C–D**). Together, our results using these chemically distinct agonists indicated that STING activation was the key factor responsible for the observed M2-to-M1 macrophage re-education.

**Figure 4:**
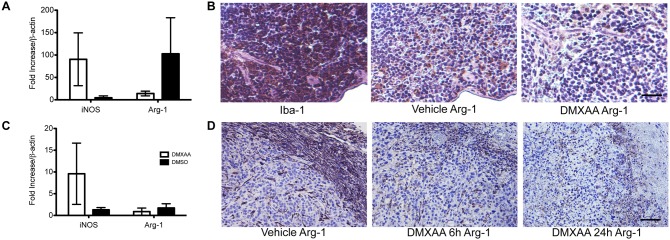
Evidence of DMXAA-mediated macrophage repolarization *in vivo*. (**A**) Spleen lysates from mice treated with 25 mg/kg DMXAA (N = 3), versus DMSO vehicle (N = 4) demonstrated decreased Arg-1, and elevated iNOS, transcripts. (**B**) Representative histology of spleen showing Arg-1 down-regulation *in vivo* in response to DMXAA. (**C**) 344SQ-ELuc whole tumor lysates from mice treated with 25 mg/kg DMXAA (N = 3), or DMSO vehicle (N = 4), also demonstrate a DMXAA-induced drop in Arg-1 and increase in iNOS transcripts. (**D**) Representative tumor sections stained with anti-Arg-1 showing a drop in Arg-1 staining as early as 6 hours post DMXAA. Scale bars = 100 µm. Data are the mean ± SEM.

RNA transcripts from spleens of mice treated *in vivo* with DMXAA also demonstrated induction of iNOS and down-regulation of Arg-1 (**Figure 4A**), as well as diminished anti-Arg-1 immunohistochemical staining (**Figure 4B**). Importantly, subcutaneous tumor lysates also demonstrated evidence of DMXAA-mediated repolarization (**Figure 4C**), with diminished Arg-1 staining being evident as early as 6 hours post-DMXAA exposure (**Figure 4D**). In summary, these results suggest that STING activation can mediate M2-like TAM re-education.

### Discordant Effects of DMXAA on 344SQ-ELuc Subcutaneous Versus Metastatic Tumors

**Figure 5:**
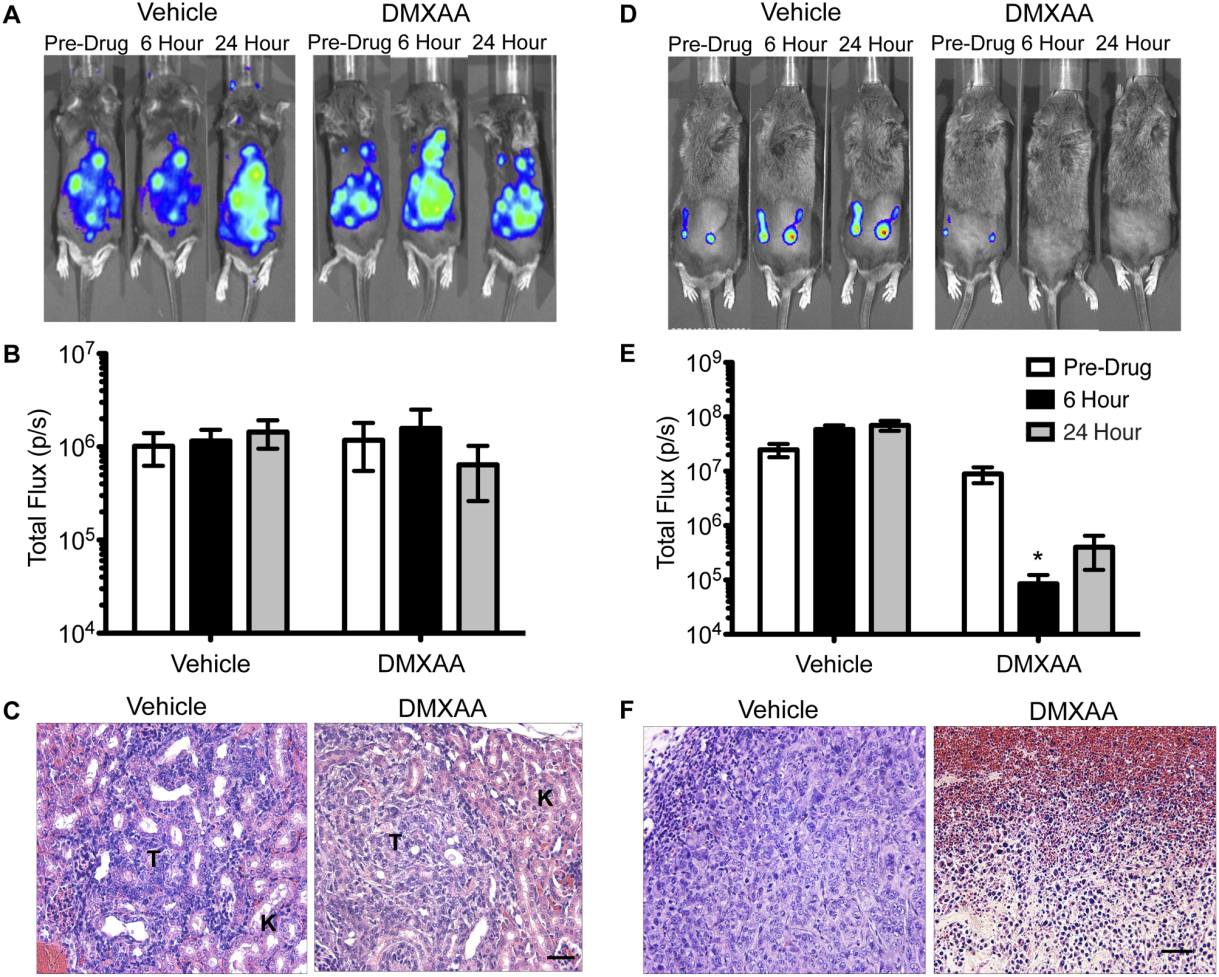
NSCLC metastases failed to show vascular disruption in response to DMXAA. (**A**) BLI of metastatic 344SQ-ELuc tumors prior to DMXAA or DMSO administration and again at 6 and 24 hours (N = 6 and 8 respectively). Whole body regions of interest (ROIs) demonstrate no loss of BLI (**B**). (**C**) Representative kidney metastases (tumor = T, kidney = K) did not show evidence of hemorrhagic necrosis after DMXAA treatment. (**D–E**) BLI of 344SQ-ELuc subcutaneous tumors at day 7 (N = 6) demonstrated a considerable drop in photon emission rates after DMXAA in mice with smaller tumors (*p<0.05), and the latter were accompanied by evidence of hemorrhagic necrosis (**F**).

**Figure 6:**
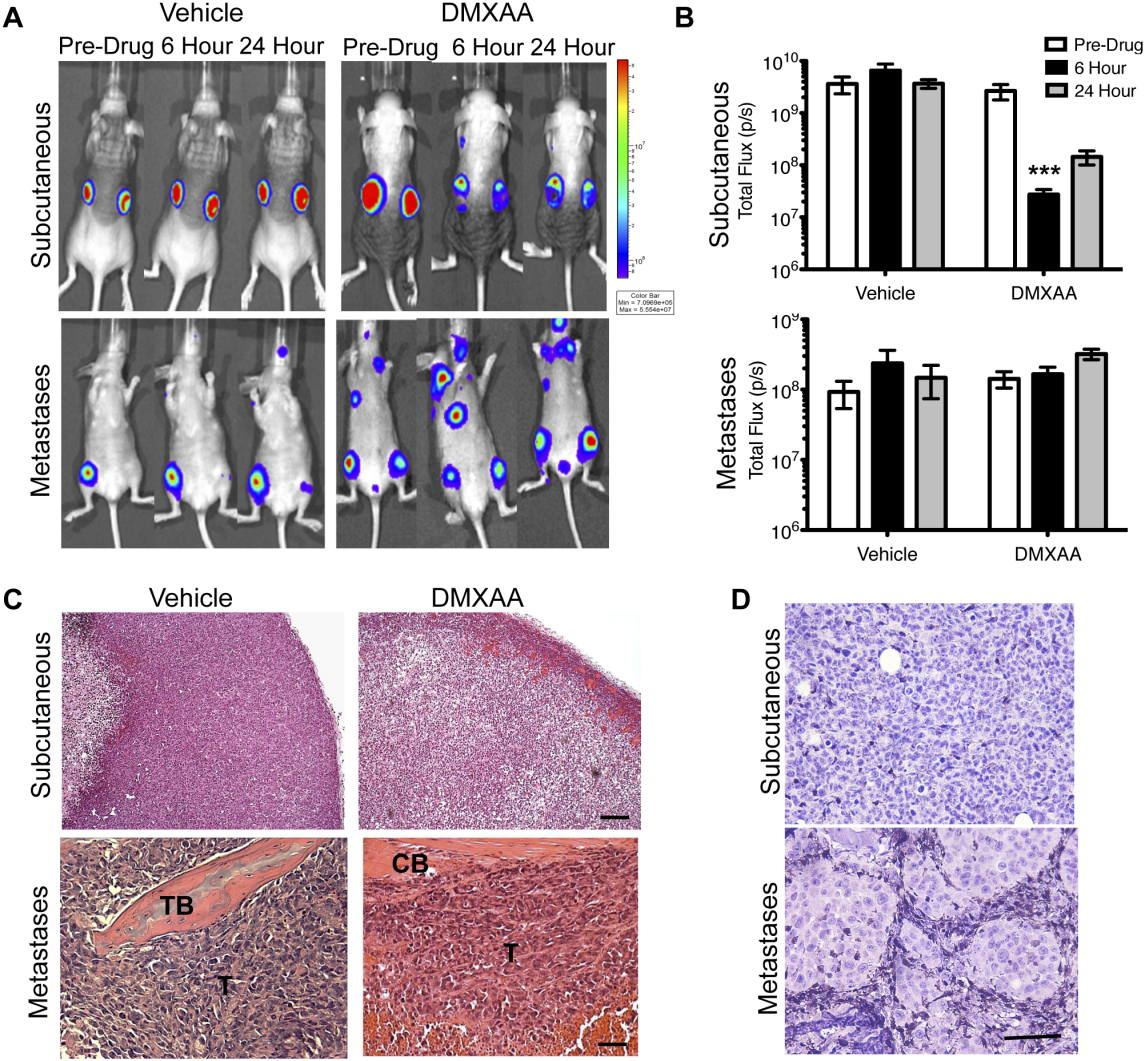
DMXAA showed differential tumor site-specific vascular disruption in a human breast cancer xenograft model. (**A**) BLI of MDA-MB-231-Luc2 subcutaneous tumors in NIH-III (*nu/nu; bg/bg*) mice 30 days post-cell inoculation, or metastases at day 21 post-cell inoculation. Mice were randomized into two groups (N = 10 each) and administered DMXAA or vehicle control, and then re-imaged at 6 and 24 hours. ROI encompassing the tumors or whole body were used to quantify photon emission rates. A significant drop in signal intensity in subcutaneous tumors treated with DMXAA, however, there was no change in light emission from DMXAA treated mice with metastatic tumors (**B**) (***p<0.001). (**C**) Representative histology of subcutaneous tumors demonstrating the presence of massive hemorrhagic necrosis in DMXAA treated mice (scale bar = 100 µm), with bone metastases (T = tumor, CB = cortical bone, TB = trabecular bone) showing only very limited regions of hemorrhage in response to DMXAA (scale bar = 50 µm). (**D**) Anti-Iba-1 staining was used to show the presence of macrophages in both subcutaneous and metastatic tumors. Data represent the mean ± SEM.

In contrast to the dramatic results obtained using 344SQ-ELuc subcutaneous tumors, DMXAA treatment of 344SQ-ELuc metastases yielded no decrease in photon emission rates (**Figure 5A–B**), with the tumors remaining histologically similar to controls after this treatment (**Figure 5C**). To confirm this effect was not peculiar to the 344SQ-ELuc cell line, we also compared the effects of DMXAA on subcutaneous versus metastatic tumors generated in MDA-MB-231-Luc2 human breast cancer xenografts. Similar to 344SQ-Eluc tumors, the MDA-MB-231-Luc2 model demonstrated dramatic hemorrhagic necrosis of subcutaneous tumors but not bone metastases (**Figure 6**).

Subcutaneous tumors grow rapidly up to ∼1 cm^3^, whereas the multiple metastases generated by 344SQ-ELuc cells are not able of reach this size owing to the lethal tumor burden that would ensue. Therefore, we evaluated the effect of DMXAA on subcutaneous 344SQ-ELuc tumors having a size (∼2 mm^3^) comparable to that of the metastases. As with the large subcutaneous tumors, DMXAA administration to mice with small subcutaneous tumors still led to ∼2-log decreases in photon emission at both 6 and 24 hours (**Figure 5D–E**). This was also accompanied by the development of pathology similar to that of the large subcutaneous tumors (**Figure 5F**). Thus, differences in tumor volume did not account for the differential effects of DMXAA on subcutaneous versus metastatic tumors.

### Primary Pulmonary Adenocarcinomas Arising in *K-Ras^la1/+^* Transgenic Mice Show No Response to DMXAA

**Figure 7:**
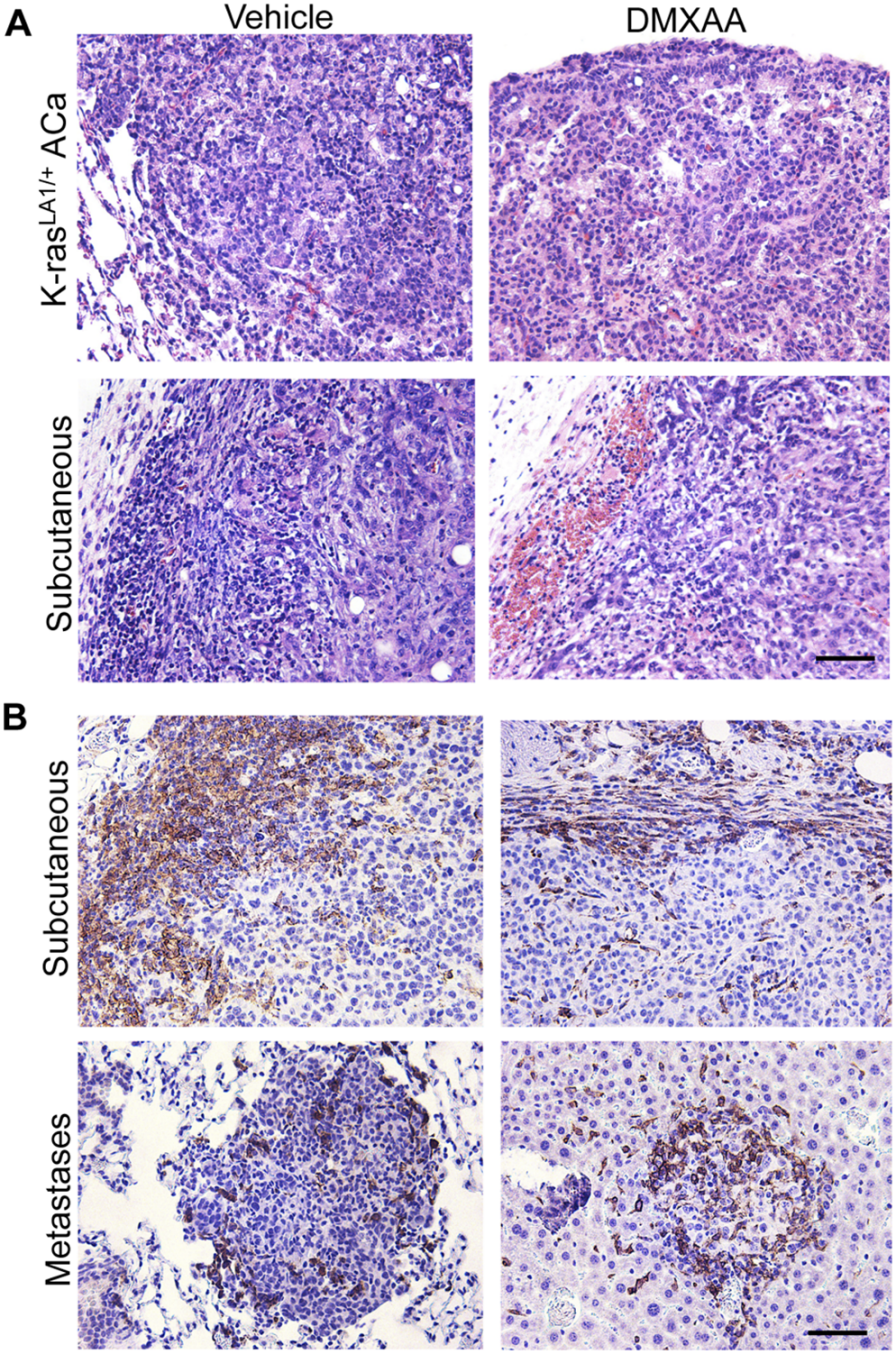
DMXAA produced no evidence of vascular disruption in spontaneous lung adenocarcinomas. (**A**) Representative histology of lung adenocarcinomas in *K-ras^LA1/+^* mice (∼150 days of age) following treatment with either vehicle (N = 3), or DMXAA (N = 6) showing no evidence of hemorrhagic necrosis with the latter. The responses of subcutaneous 344SQ-ELuc tumors (N = 6) to DMXAA injection are shown for comparison purposes. (**B**) Tumor sections stained with anti-Iba-1 demonstrated a thick rim of macrophages at the tumor periphery in 344SQ-ELuc subcutaneous day 14 (top left) and day 7 (top right) tumors. In contrast, there were variable and much lower levels of macrophage infiltration within 344SQ-ELuc metastases, for example, in lung (bottom left) or liver (bottom right). Scale bar = 100 µm (F, G), 50 µm (C, H). Data represent the mean ± SEM.

We next evaluated the effects of DMXAA on spontaneously arising primary NSCLCs in the *K-ras^LA1/+^* transgenic model [26] of lung cancer. Again, in contrast to the dramatic results seen with the subcutaneous tumors, DMXAA treatment of *K-ras^LA1/+^* mice (∼150 days old) produced no discernable histological effect on the lung adenocarcinomas (**Figure 7A**). It was possible that the variable level of macrophage infiltration amongst different tumor sites may have accounted for the inconsistent responses to DMXAA. Thus, while primary *K-ras^LA1/+^* lung tumors have even fewer numbers infiltrating macrophages (**Figure 1C**) than systemic metastases, subcutaneous tumors show abundant infiltrates of macrophages and neutrophils (data not shown) in the tumor periphery (**Figure 7B**). Thus, the level of TAM infiltration could be one potential variable determining whether DMXAA will cause vascular disruption and hemorrhagic necrosis.

### Primary NSCLC Tumors Exhibit Increased Evans Blue Dye Permeability

**Figure 8:**
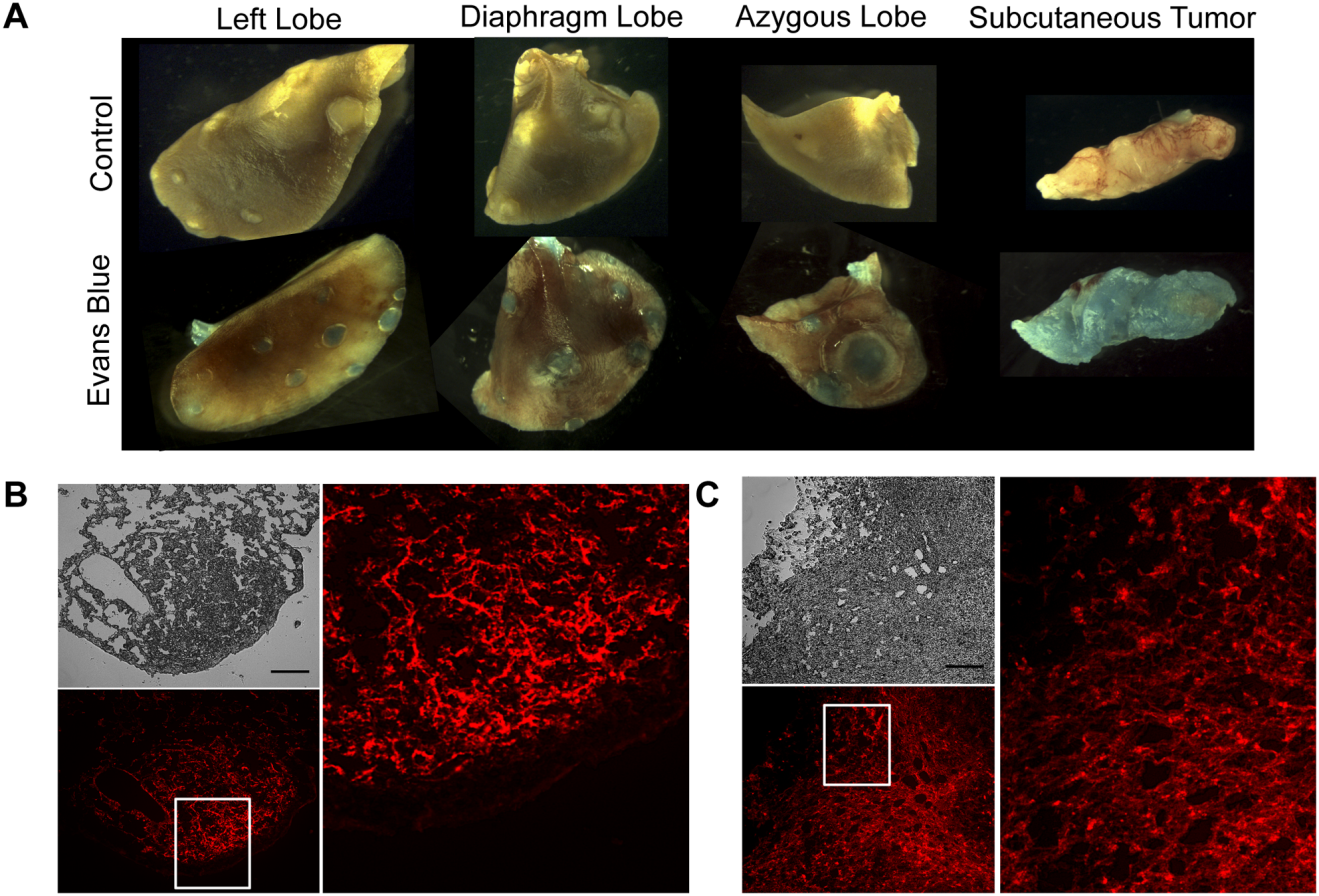
No differences in Evans blue permeability was present between primary lung neoplasms and subcutaneous tumors. (**A**) Whole-mount images of adenoma- and adenocarcinoma-bearing *K-ras^LA1/+^* lungs and 344SQ-ELuc subcutaneous tumors following Evans Blue dye injection, or PBS control. (**B**) Fluorescence images of lung sections (phase contrast shown in grey, and reflected light in red) and subcutaneous tumor sections in (**C**) (Scale bar = 100 µm). The dye was able to extravasate in both tumor locations, suggesting that lack of permeability to small molecules did not account for the failure of DMXAA to disrupt the vasculature of primary lung neoplasms.

DMXAA is known to target the unstable, leaky vasculature of tumors [3], [7]–[9], thus, we investigated whether diminished permeability might be a potential factor rendering the primary tumor vasculature resistant to DMXAA. Using a modified Miles assay, we injected mice with the Evans blue dye to look for interstitial leakage in *K-ras^LA1/+^* lung adenomas and adenocarcinomas, and in 344SQ-ELuc subcutaneous tumors. Primary lung neoplasms (adenomas and adenocarcinomas) demonstrated similar dye permeability as subcutaneous tumors (**Figure 8A**). Frozen sections of *K-ras^LA1/+^* lung (**Figure 8B**), and subcutaneous tumors (**Figure 8C**) demonstrated similar dye leakage into tumors at both sites (see also **Figure S5**). Hence, the possibility that vessel impermeability to small molecules such as DMXAA was a factor accounting for the inability of this agent to cause vascular disruption in the primary tumors was discounted.

### Subcutaneous and Metastatic 344SQ-Eluc Tumors Exhibit Differences in Vasculature Structure

**Figure 9:**
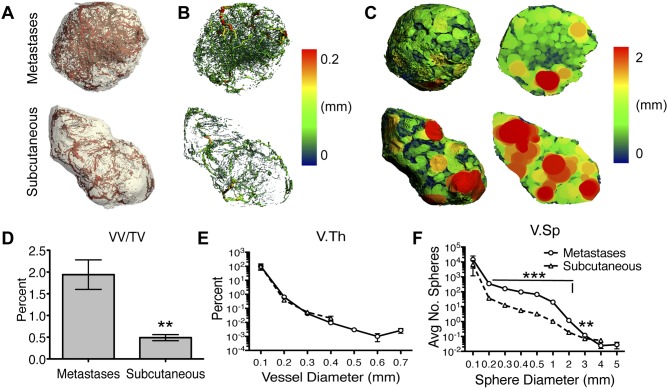
Differences in vascular structure were present between subcutaneous 344SQ-ELuc tumors and metastatic *p53^R172HΔg/+^ K-ras^LA1/+^* NSCLC. (**A**) Micro-CT 3D rendering of tumors from Microfil-perfused mice harboring 344SQ-ELuc subcutaneous tumors or *p53^R172HΔg/+^ K-ras^LA1/+^* lung adenocarcinoma-derived metastases (N = 6). (**B**) Vessel thickness (V.Th) represented by heat-map, red vessels ≥0.2 mm diameter (**C**) Vessel separation (V.Sp) represented by the maximal sphere-filling model (red spheres indicating a diameter of ≥2 mm between vessels). Full view and cut-plane through tumor center demonstrates markedly increased avascularity of subcutaneous tumors as compared to metastases. (**D**) Quantification of vessel density (VV/TV) confirms significantly less vessels present in subcutaneous tumors (**p<0.01). (**E**) Distribution of V.Th is represented as a percentage of total vessels, indicates similar pattern in both tumor locations (Log10 scale). (**F**) Distribution of average number of spheres as an indicator of V.Sp demonstrated significantly fewer small-diameter spheres in subcutaneous tumors (***p<0.001), indicative of ischemic and/or necrotic regions (Log^10^ scale). Data are represented as the mean ± SEM.

Since differences in tumor vascular bed structure could be a factor accounting for the differential responses to DMXAA, we compared subcutaneous 344SQ-ELuc tumors and *p53^R172HΔg/+^ K-ras^LA1/+^* lung adenocarcinoma-derived spontaneous metastases using a 3D-microcomputed tomography (micro-CT) imaging quantification method we previously described [22]. Interestingly, 3D renderings of 344SQ-ELuc subcutaneous tumors and *p53^R172HΔg/+^ K-ras^LA1/+^* metastases displayed significant differences with respect to overall appearance (**Figure 9A**), vessel thickness (**Figure 9B**), and most strikingly, with respect to internal avascular areas, as demonstrated by a sphere-filling computational technique [22] (**Figure 9C**). Quantification of vessel parameters demonstrated a significant drop in vessel density (VV/TV) (**Figure 9D**) as well as overall vessel number (V.N) and connectivity (Conn.D) in subcutaneous tumors (**Figure S6**). Interestingly, there was a similar pattern in vessel thickness (V.Th) when compared as a percentage of vessels present, although the metastases had larger-diameter vessels (**Figure 9E**). Vessel separation (V.Sp), on the other hand, demonstrated a significant difference between the two different tumor sites, with subcutaneous tumors having markedly less small-diameter spheres (an indication of well vascularized areas) (**Figure 9F**), thus confirming the presence of larger avascular/ischemic areas in the subcutaneous tumors (as visualized by the red spheres in **Figure 9C**). Thus, it was plausible that differences in the structure of the tumor vascular network between the different tumor sites might also contribute to the differential responses to DMXAA.

## Discussion

Our findings using both DMXAA and 2′3′-cGAMP suggest that STING activation was the common factor leading to M2 macrophage re-polarization, a process that undoubtedly played a role in mediating the vascular disrupting effects of DMXAA we observed on the subcutaneous 344SQ-ELuc tumors. Interestingly, however, we found that the vascular disrupting effects of DMXAA on subcutaneous tumors did not extend to either the 344SQ-ELuc metastases obtained following intracardiac injection of these cells, or to the spontaneously arising tumors in the *K-ras^LA1/+^* GEMM model of NSCLC. Indeed, the majority of successful pre-clinical studies evaluating the utility of DMXAA were carried out in subcutaneous tumor models, with relatively few studies examining the effects of DMXAA on tumors in other anatomical sites [37], [38]. Echoing our results, experiments employing a different vascular disrupting drug, flavone acetic acid (FAA), vessel disruption was seen in subcutaneous tumors, but not systemic tumors [39]. Thus, although the inability of DMXAA to activate human STING provided an obvious reason for failure of DMXAA in human cancer trials [20], [21], our results nevertheless suggest that vascular disruption might not occur in either primary or metastatic human NSCLC if human STING agonists were administered. With regard to correcting this defect, considerable efforts are now underway involving the development of stable cyclic dinucleotide analogs that will allow human STING activation [40].

Differences in the density of TAM infiltration amongst different tumor sites may have been one of several factors accounting for the differential effects of DMXAA on subcutaneous versus 344SQ-ELuc metastases. Supporting the key role of infiltrating TAMs, we found that their depletion in subcutaneous 344SQ-ELuc tumors prevented DMXAA-induced intra-tumoral hemorrhagic necrosis. In the case of 344SQ-ELuc subcutaneous tumors, TAMs were present as a dense rim at the tumor periphery and were thus well positioned to support DMXAA-induced vascular disruption via the production of pro-inflammatory mediators. There were extensive regions of ischemic necrosis invariably present within the subcutaneous 344SQ-ELuc tumors, signifying the availability of macrophage activating environmental factors such as hypoxia and the Toll-like receptor (TLR) 4-activating protein, high mobility group, HMGB1 protein [41]. Thus, in addition to quantitative differences in macrophage infiltration density between tumor sites, there may have been substantial qualitative differences between the TAMs at different tumor sites.

Macrophages are inherently plastic, with the M1- and M2-polarized phenotypes representing the extremes of a spectrum [33], [42], [43]. Although TAMs are often M2-like, there is now evidence that such TAMs can be re-polarized towards an M1 phenotype that can inhibit tumor growth [31], [32], [44], [45]. Herein, we show that the STING agonists DMXAA and 2′3′-cGAMP are both able to repolarize M2-polarized marrow-derived macrophages *in vitro*, and we also provide evidence that DMXAA is able to shift M2-like macrophages towards an M1-like phenotype *in vivo*. The latter results were in agreement with a recent study reporting an M2-like to M1 shift in TAM populations in response to DMXAA treatment that was also were accompanied by an anti-tumor effect [46]. Intra-tumoral TAM repolarization would provide a source of pro-inflammatory cytokines and chemokines, and reduce the levels of vascular endothelial growth factor, effects that could contribute towards either vascular disruption, or stabilization, respectively. TAM re-education with STING agonists may play a role in other important processes, including promotion of anti-tumor adaptive immune responses that are dependent on type I interferons and dendritic cell activation [47], [48].

The sensitivity of tumor vasculature to DMXAA is thought to be due to the immature and irregular vascular patterning within tumors [2], [7]. To produce subcutaneous tumors, large numbers of cancer cells are implanted and these divide rapidly, rendering them sensitive to chemotherapeutic agents [49]. Growth of cells introduced in this manner may outstrip the angiogenic capacity of the host, leading to the development of large regions of ischemia and necrosis that can promote macrophage infiltration and activation. In both the 344SQ-ELuc metastases and the autochthonic NSCLC tumors there were no areas of necrosis, in contrast, subcutaneous 344SQ-ELuc tumors contained large necrotic regions and were densely populated with macrophages. It is possible that the ischemia and necrosis that typifies subcutaneous tumors renders their vessels more susceptible to DMXAA. In addition, it is plausible that compared to vessels derived from other vascular beds, dermal vasculature-derived tumor vessels are inherently unstable, and hence more vulnerable to DMXAA. Co-option of mature vessels may be a feature of both systemic metastases and primary lung adenocarcinomas, and such vessels, like other normal vascular beds in the animal, would be predicted to be refractory to the vascular disrupting effects of DMXAA. Our finding of structural differences in the vasculature between tumors at different anatomical sites lends some support the latter notion, an idea that was reinforced by the apparent inability of clodronate liposomes to cause macrophage deletion in 344SQ-ELuc metastases. Indeed, the latter observation provided a functional indication of structural differences between subcutaneous tumors and metastases. In summary, and as depicted in **Figure S7**, the features of the subcutaneous 344SQ-ELuc tumor vasculature may render them susceptible vascular disrupting effects of DMXAA. In contrast, the 344SQ-ELuc metastases, having arisen not only within different vascular beds, but importantly, from a very much smaller initial tumor colonizing cell numbers, contain vessels that are resistant to this agent.

In view of the evidence that DMXAA, acting via its effects on TAMs and/or dendritic cells, has the capacity to augment adaptive cytotoxic T cell anti-tumor activity, reviewed in [48], it would be of considerable interest to determine whether chronic administration of STING agonists might similarly lead to spontaneous immunity against 344SQ-ELuc metastases and primary lung adenocarcinomas. The fact that 344SQ-ELuc metastases do not undergo hemorrhagic necrosis in response to DMXAA would actually be of benefit in this setting, since it would allow anti-tumor immunity to be readily quantified via changes in photon emission rates.

Recent studies suggest GEMMs may be able to more faithfully mimic their human counterparts, not only with respect to genetic alterations, but also in their ability to predict responses to therapy [28], [29], [50], [51]. Thus, while primary orthotopic or subcutaneous models are useful for initial drug screening, new agents also need to be evaluated using a range of preclinical models before their use in humans is contemplated [49]. Interestingly, we found that metastases resulting from intra-cardiac injection of 344SQ-ELuc cells failed to respond to DMXAA, suggesting that GEMM-derived cell lines might serve as effective surrogates for the corresponding slowly growing autochthonous cancers. Regardless of whether or not STING agonists are ultimately found to cause vascular disruption in human cancer, the potential for such agents to repolarize TAMs will render them useful additions to the anti-cancer armamentarium.

## Materials and Methods

### Mice

Male 129/Sv mice (6–12 week old) were used for syngeneic tumor studies. Transgenic mice harboring the *p53^R172HΔg/+^* and *K-ras^LA1/+^* mutations were kindly provided Dr. G. Lozano (University of Texas) [23], [26], [52]. Mice were maintained on standard mouse chow (Pico-Vac Lab Mouse Diet #5062), and housed in a specific pathogen-free barrier facility with ethics approval from the University of Calgary Animal Care Committee (protocols M10063 and M08112) and in accordance with Canadian Council on Animal Care guidelines.

### Cell Lines and Culture

The *p53^R172HΔg/+^ K-ras^LA1/+^* lung adenocarcinoma subcutaneous metastasis-derived 344SQ NSCLC (male) cell line, generously provided by Dr. J. Kurie (University of Texas) [23], [24], was transfected with a EGFP-Luc2 fusion gene dual reporter as previously described [22], [25]. 344SQ-EGFP-Luc2 (designated herein as 344SQ-ELuc) cells were cultured in RPMI 1640 (Invitrogen), supplemented with 10% FBS, 100 U/ml penicillin, 100 µg/ml streptomycin and 0.8 mg/ml geneticin (Invitrogen) in a 37°C 5% CO_2_ humidified incubator. Bone marrow-derived macrophages (BMDM) were collected from 129/Sv mice (N = 3). Cells were resuspended in DMEM (Lonza) supplemented with 100 U/ml penicillin, 100 µg/ml streptomycin, 10 mM sodium pyruvate, 20 mM *L*-glutamine (Invitrogen), 10% FBS (Hyclone) and 20% L929 cell conditioned media. BMDM were plated onto 100 mm bacterial plates and incubated at 37°C in a 5% CO_2_ humidified atmosphere.

### Tumor Generation and Drug Treatments

To generate subcutaneous tumors, 5×10^5^ 344SQ-ELuc cells in 100 µl PBS were injected in both posterior flanks of mice. Metastatic tumors were generated via intracardiac injection of 1×10^4^ 344SQ-ELuc cells in 100 µl PBS into the left ventricles (LV) of mice anaesthetized with 100 mg/kg ketamine and 6 mg/kg xylazine, given intra-peritoneally (i.p.). Tumor growth was monitored every 2–4 days via BLI. Mice were shaved to minimize light signal attenuation. Once tumors were established (day 10 for systemic metastases; day 7 or day 14 for subcutaneous tumors), mice were given 25 mg/kg of DMXAA (D5817, Sigma-Aldrich), or DMSO vehicle by i.p. injection. BLI was carried out at 6 and 24 hours as previously described [22]. *K-ras^LA1/+^* mice, aged ∼150 days received 25 mg/kg DMXAA or DMSO and were sacrificed 6 hours later. TAM depletion was carried out via i.p. injection of Clodrolip (2 mg/20 g mouse) starting at day −1 prior to tumor cell inoculation, and was maintained with 1 mg/20 g mouse given every 3 days for the duration of the experiment. Empty liposomes (EL) served as the controls [27].

### Breast Cancer Metastases Model

The human breast cancer cell line MDA-MB-231 was previously transfected with a dual reporter (an EGFP-Luc2 fusion protein) [25]. MDA-MB-231-EGFP-Luc2 cell (designated herein as MDA-MB-231-Luc2) were cultured in Dulbecco’s modified Eagle medium (DMEM; Invitrogen), supplemented with 10% fetal bovine serum (FBS), 100 U/ml penicillin, 100 µg/ml streptomycin and 0.8 mg/ml geneticin (Invitrogen) in a 37°C 5% CO_2_ humidified incubator. Athymic-beige (NIH-III) female mice (4–5 week old) were purchased from Charles River Laboratories (St. Constant, QC) and subcutaneous tumors (analyzed at day 21) were generated by intradermal implantations of 5×10^5^ cells in 100 µl PBS over both the right and left posterior flanks. Metastatic tumors (analyzed at day 30) were generated via LV injection of 2×10^5^ cells in 100 µl PBS into anesthetized mice (100 mg/kg ketamine and 6 mg/kg xylazine, given i.p.). Knees were fixed at 4°C in 4% paraformaldehyde (PFA) for 7 days, followed by decalcification in 14% ethylenediaminetetraacetic acid (EDTA) prior to paraffin embedding and sectioning.

### Histopathology

Tumors were fixed in 10% neutral buffered formalin (NBF), and lungs were inflated with 10% NBF via a tracheal cannula. Tissues were processed for paraffin embedding and sectioned at 4 µm prior to staining with hematoxylin and eosin (H&E). For immunohistochemistry, anti-Iba1, 1∶500 (Wako) anti-Arginase-1, 1∶100 (Santa Cruz Biotechnology) antibodies were used, with Vectastain IgG (ABC kit, Vector Laboratories) secondary with DAB substrate development (Sigma-Aldrich). Hematoxylin served as the counterstain.

### Cytotoxicity Assay

Assays for DMXAA and Clodrolip cytotoxicity used 3-(4,5-dimethyl-2-thialolyl)-2,5-diphenyl-2H-tetrazoliumbromide (MTT reagent; Sigma, St. Louis, MO) as per instructions provided by the manufacturer. Absorbance values were determined on a Multiskan Ascent Microplate Reader (Thermo Labsystems, Finland).

### Flow Cytometry

BMDM were collected from Clodrolip or EL treated mice and suspended in 2% FBS/PBS and incubated with Fc Block (anti-CD16/CD32), followed by anti-CD11b (BD), and anti-F4/80 antibodies (eBioscience). Samples were run on a FACScalibur equipped with CellQuest software (BD) and quantified using FlowJo software (TreeStar).

### Reverse Phase Protein Array (RPPA)

M2-polarized (40 ng/ml IL-4 for 48 hours, N = 3) macrophages were treated with 20 µg/ml DMXAA or DMSO vehicle for 30 min. Cells were then lysed and protein denatured in SDS buffer and samples sent for RPPA analysis (RPPA Core Facility, University of Texas, MD Anderson Cancer Center, Houston, TX). Differential abundance of various proteins and/or their phosphorylation status in response to DMXAA was assessed.

### Vascular Perfusion and Micro-computed Tomography (micro-CT)

Mice with 344SQ-ELuc subcutaneous tumors, and aged *p53^R172HΔg/+^ K-ras^LA1/+^* mice (∼200 day old) with metastases were used to evaluate the tumor vasculature. Prior to sacrifice, mice were given a sub-lethal i.p. dose of ketamine (100 mg/kg) plus xylazine (6 mg/kg). Cardiac perfusions with Microfil were as previously described [22], [53]. Excised tumors were scanned using a µCT35 (Scanco Medical AG) at a nominal resolution of 10 µm (55 kVp, 145 µA, 200 ms integration time, 1000 proj/180 degrees, 20.5 mm diameter field of view, 2048×2048 reconstruction matrix, cone-beam reconstruction) as previously described [22].

### Vascular Permeability

Vessel permeability in mice with subcutaneous 344SQ-ELuc tumors and aged *K-ras^LA1/+^* mice (∼200 days old) was examined using a modified Miles Assay. Mice were given a 4 µl/g dose of 2% Evans Blue dye (Sigma) made up in 0.9% saline by i.p. injection. After 5 hours, mice were perfused with PBS followed by 4% PFA using a Masterflex C/L perfusion pump (Cole-Parmer). Tissues were harvested and lungs were inflated via a tracheal cannula prior to sinking them in 30% sucrose/PBS and OCT embedding (Tissue-Tek). Whole tissues were imaged on a Stemi SV 11 dissection microscope (Zeiss). Samples were cryosectioned at 10 µm and imaged on an Axio Imager A2 (Zeiss) under a Cy5 filter.

### Macrophage Polarization and Supernatant Cytokine Assay

BMDM were seeded in 6-well plates at 2×10^6^ cells/well and polarized for 48 hours with the addition of 50 ng/ml LPS (List Biological Labs) and 50 ng/ml IFNγ (Cedarlane) for M1, or 40 ng/ml IL-4 (R&D Systems) for M2 at 37°C in a 5% CO_2_ humidified atmosphere. Cells were re-plated in triplicate in 96-well plates, 8×10^5^ cells/well, in media containing 20 µg/ml DMXAA or DMSO control for 24 hours. Supernatants were subjected to a mouse 32-plex cytokine/chemokine discovery array (Luminex) (EVE Technologies, Calgary, Alberta).

### RT-PCR

Subcutaneous tumors and control spleens were snap frozen in liquid N_2_ prior to being homogenized in QIAzol lysis reagent (Qiagen). M1 and M2 polarized macrophages were treated with 20 µg/ml DMXAA (or dose response) or DMSO vehicle for an additional 24 hours. In addition, M2 polarized macrophages were treated with 20 µg/ml or 40 µg/ml 2′3′-cGAMP (InvivoGen) in the presence of Lipofectamine-2000 (LF2000, Invitrogen). Cells were lysed with QIAzol lysis reagent (Qiagen) and RNA was extracted with chloroform and isopropanol. To make cDNA, 1 µg of RNA was treated with DNAse (Promega) followed by RT-PCR with 10 mM dNTPs, random primers (Roche) and Superscript II reverse transcriptase (Invitrogen). Real-time PCR of cDNAs was carried out on the LightCycler using the LightCycler FastStart DNA MasterPLUS SYBR Green kit (Roche). Data were normalized to β-actin mRNA. Primer sequences were as previously described [44], [54], [55].

### Statistical Methods

Samples were compared using a student’s *t*-test, with Welch’s correction on samples with unequal variance. *P* values of <0.05 were considered significant.

## Supporting Information

Figure S1 **Intracardiac injection of 344SQ-ELuc cells generated a spectrum of systemic metastases.** BLI of mice after intracardiac injection of 344SQ-ELuc cells at day 4, 7, 10 and 14. Dorsal view (**A**) and ventral view (**B**). Histology revealed cells growing within the heart muscle (**C**), likely as a result of the cell leakage at the LV injection site. Other common locations for tumor growth included lung (**D**), adrenal gland (**E**), kidney (**F**), visceral fat pad (**G**), and less frequently liver (**H**), and spleen (**I**). Scale bars = 100 µm (F–G) and 50 µm (C, D, E, H, I).
(TIF)

Figure S2 **Clodrolip depletion of macrophages prior to 344SQ-ELuc subcutaneous implantation modestly inhibited tumor formation.** (**A**) BMDM from Clod or EL treated mice (N = 3) at day 10 were collected and stained for CD11b and F4/80 and subjected to FACS, demonstrating an approximately 50% drop in CD11b^+^ F4/80^+^ macrophages as quantified in (**B**) (***p<0.001, gated for monocytes). BLI growth rates of subcutaneous (**C**; N = 5) and LV (**E**; N = 8) 344SQ-ELuc tumors in 129/Sv mice demonstrated a modest lag in tumor formation in subcutaneous tumors, but produced no difference in the development of 344SQ-ELuc metastases following clodronate-mediated macrophage depletion, with tumors still developing regardless of inoculation route (**D, F**).
(TIF)

Figure S3 **DMXAA did not show direct toxicity against NSCLC cells** ***in vitro*** **.** *In vitro* cytotoxicity of DMXAA on 344SQ-ELuc cells obtained via MTT assays. Data are represented as the mean ± SEM.
(TIF)

Figure S4 **Polarization of BMDM.** (**A**) BMDM were treated with 50 ng/ml LPS and 50 ng/ml IFNγ for M1 polarization, or 40 ng/ml IL-4 for M2 polarization. After 48 hours, RNA transcript levels confirmed polarization had occurred by showing upregulation of iNOS in M1 macrophages, and Arg-1 in M2 macrophages. (**B**) Reverse phase protein array analysis of M2 macrophages treated with 20 µg/ml DMXAA for 30 min showed up-regulation of phosphorylated p65 (N = 3, ***p≤0.001).
(TIF)

Figure S5 **Vessel permeability of subcutaneous versus metastatic tumors.** (**A**) Phase-contrast and corresponding fluorescence images of control tissues demonstrate Evans blue dye outlining the vessels in the lung and kidney, consistent with low levels of background dye leakage. The vessels in the brain, however, showed no leakage, consistent with the dyes inability to cross the blood brain barrier. (**B**) Representative images of Evans Blue dye leakage in three different lung tumors, and (**C**) subcutaneous tumors. Scale bar = 100 µm.
(TIF)

Figure S6 **Quantification of vessel parameters of subcutaneous versus metastatic tumors.** (**A**) There was decreased vessel connectivity in subcutaneous 344SQ-ELuc tumors compared to primary adenocarcinoma-derived *p53^R172HΔg/+^ K-ras^LA1/+^* metastases as well as a decrease in vessel number (**B**) (not significant). Vessel surface area/vessel volume (**C**), however, was similar between the tumor sites indicating that the dimensions of the vessels present were comparable. Data represented as mean ± SEM.
(TIF)

Figure S7 **Summary of DMXAA effects in the murine NSCLC models.** Induction of M2-like TAM repolarization towards an M1-like phenotype by DMXAA accompanied the rapid onset of hemorrhagic necrosis of subcutaneous tumors. In contrast, DMXAA did not exhibit vascular disrupting effects on either syngeneic metastases or spontaneously arising NSCLC in tumors in *Kras^LA1^* GEMM model.
(TIF)

Table S1 **DMXAA induction of cytokines and chemokines in M1 versus M2 polarized macrophages.** Polarized BMDM (N = 3) were treated with DMXAA or DMSO control and serums were analyzed via cytokine array. Average values are shown.
(DOCX)
