# Supplementary figures and images for: DMXAA Causes Tumor Site-Specific Vascular Disruption in Murine Non-Small Cell Lung Cancer, and like the Endogenous Non-Canonical Cyclic Dinucleotide STING Agonist, 2′3′-cGAMP, Induces M2 Macrophage Repolarization
Source: PLoS One. 2014 Jun 18;9(6):e99988. doi: 10.1371/journal.pone.0099988 (PMC4062468; doi:10.1371/journal.pone.0099988)

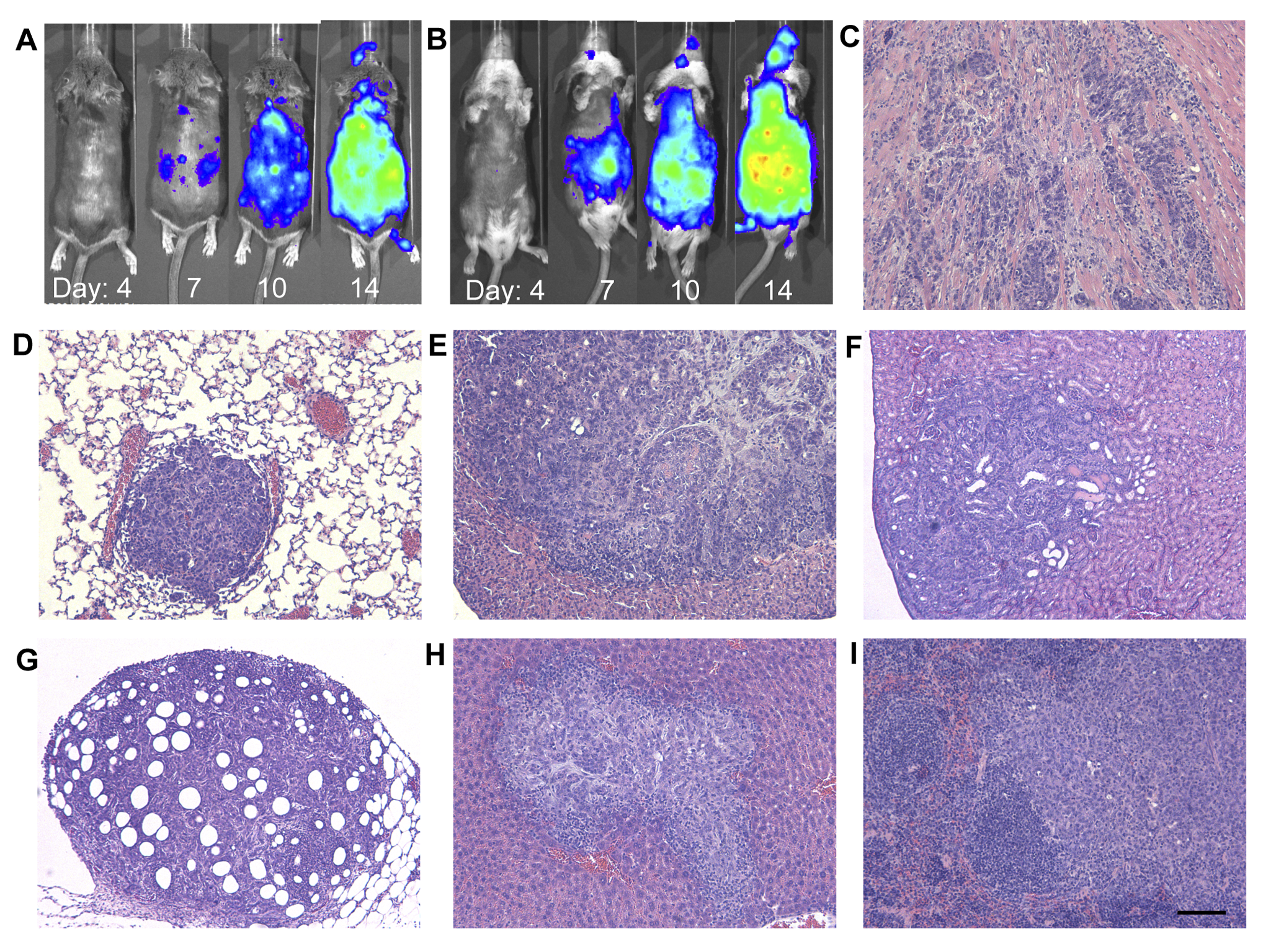

Supplement: Figure S1 — Intracardiac injection of 344SQ-ELuc cells generated a spectrum of systemic metastases. BLI of mice after intracardiac injection of 344SQ-ELuc cells at day 4, 7, 10 and 14. Dorsal view (A) and ventral view (B). Histology revealed cells growing within the heart muscle (C), likely as a result of the cell leakage at the LV injection site. Other common locations for tumor growth included lung (D), adrenal gland (E), kidney (F), visceral fat pad (G), and less frequently liver (H), and spleen (I). Scale bars = 100 µm (F–G) and 50 µm (C, D, E, H, I). (TIF) [file pone.0099988.s001.tif]

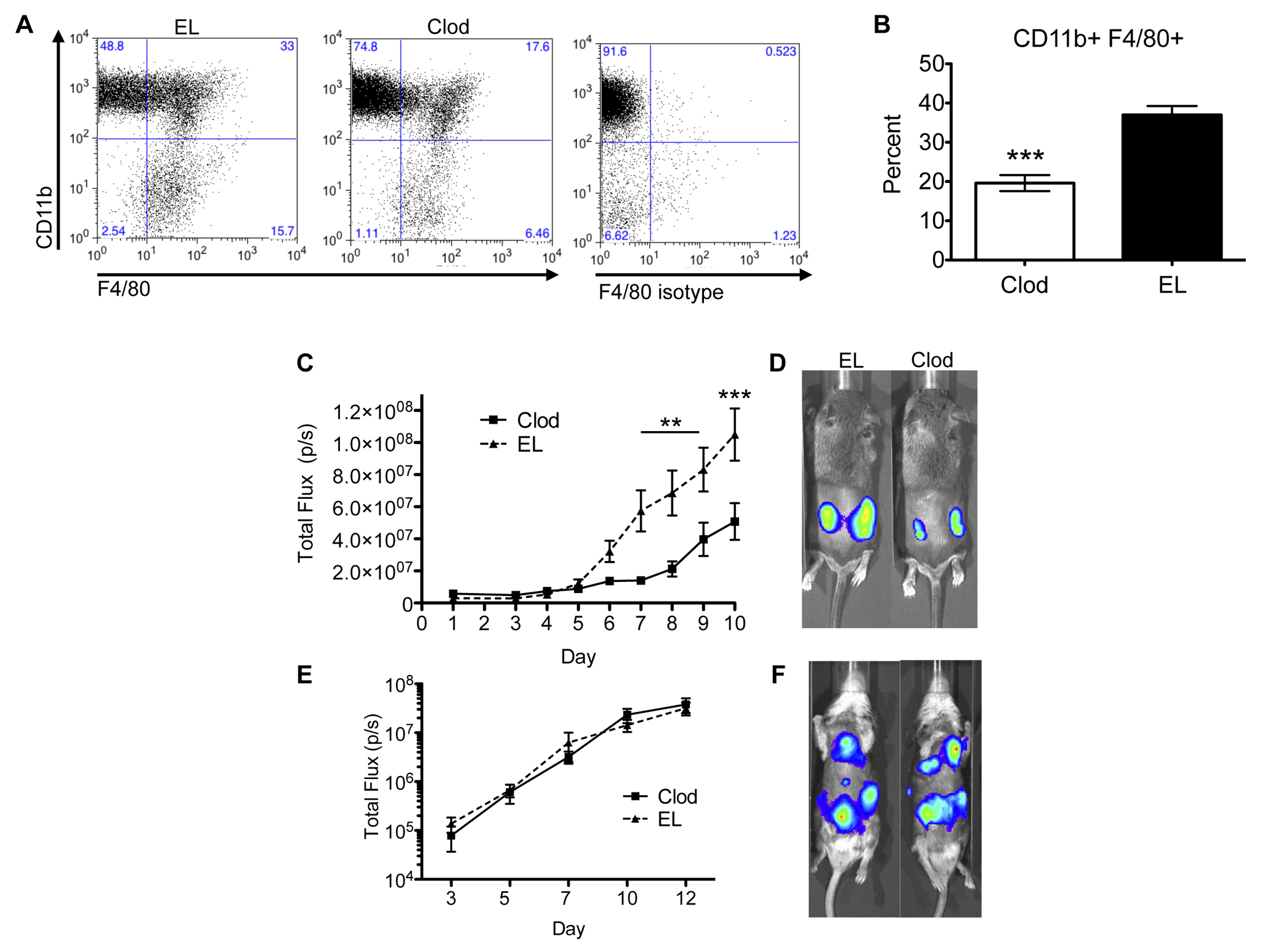

Supplement: Figure S2 — Clodrolip depletion of macrophages prior to 344SQ-ELuc subcutaneous implantation modestly inhibited tumor formation. (A) BMDM from Clod or EL treated mice (N = 3) at day 10 were collected and stained for CD11b and F4/80 and subjected to FACS, demonstrating an approximately 50% drop in CD11b+ F4/80+ macrophages as quantified in (B) (***p<0.001, gated for monocytes). BLI growth rates of subcutaneous (C; N = 5) and LV (E; N = 8) 344SQ-ELuc tumors in 129/Sv mice demonstrated a modest lag in tumor formation in subcutaneous tumors, but produced no difference in the development of 344SQ-ELuc metastases following clodronate-mediated macrophage depletion, with tumors still developing regardless of inoculation route (D, F). (TIF) [file pone.0099988.s002.tif]

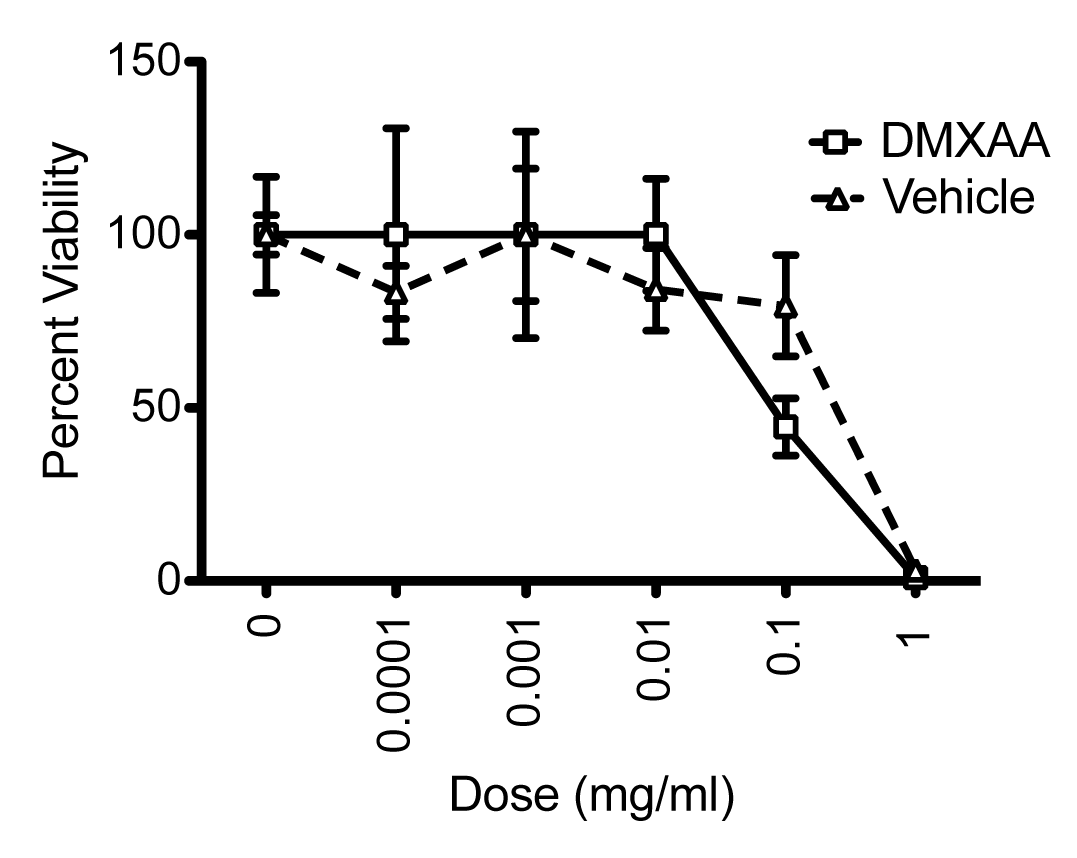

Supplement: Figure S3 — DMXAA did not show direct toxicity against NSCLC cells in vitro . In vitro cytotoxicity of DMXAA on 344SQ-ELuc cells obtained via MTT assays. Data are represented as the mean ± SEM. (TIF) [file pone.0099988.s003.tif]

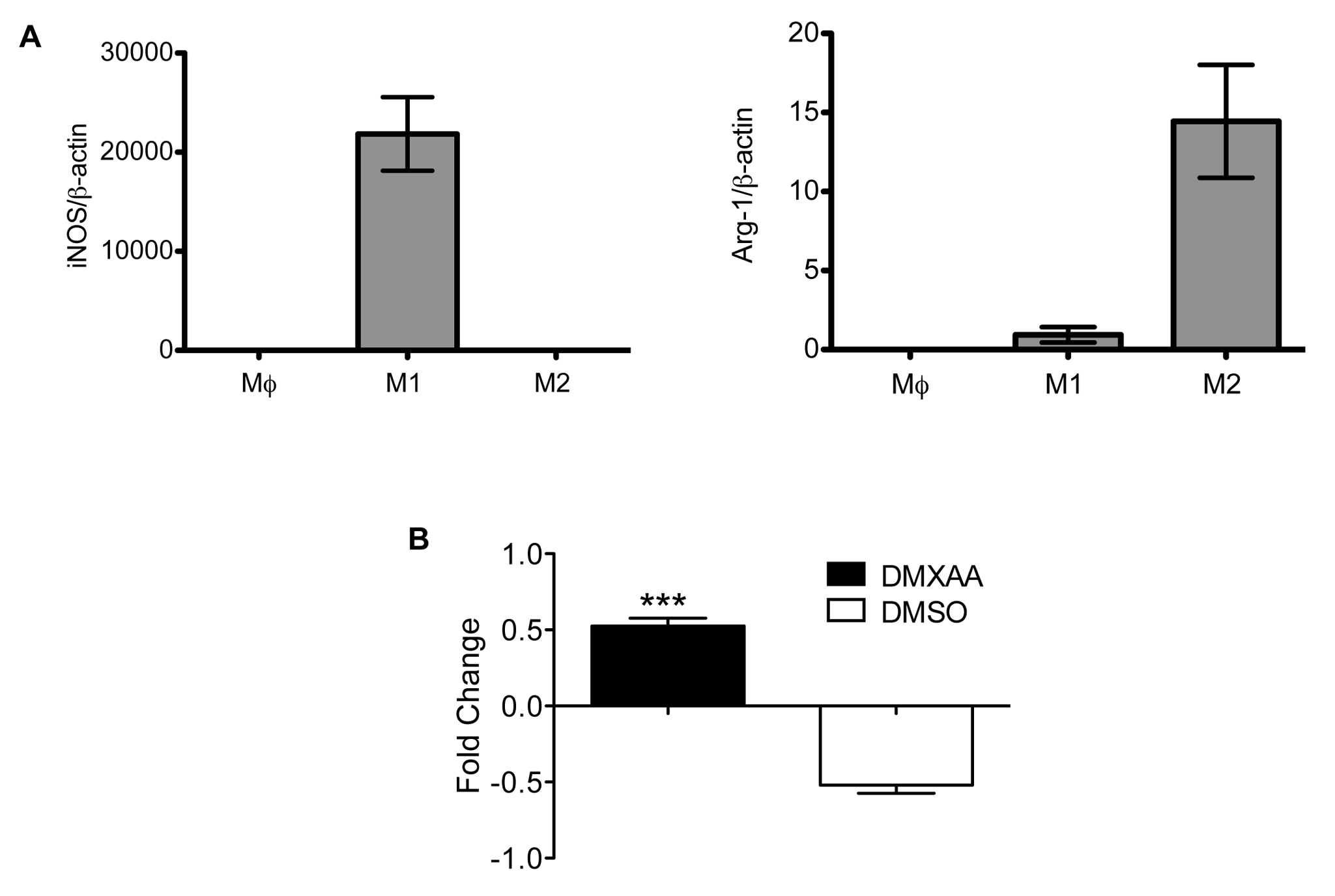

Supplement: Figure S4 — Polarization of BMDM. (A) BMDM were treated with 50 ng/ml LPS and 50 ng/ml IFNγ for M1 polarization, or 40 ng/ml IL-4 for M2 polarization. After 48 hours, RNA transcript levels confirmed polarization had occurred by showing upregulation of iNOS in M1 macrophages, and Arg-1 in M2 macrophages. (B) Reverse phase protein array analysis of M2 macrophages treated with 20 µg/ml DMXAA for 30 min showed up-regulation of phosphorylated p65 (N = 3, ***p≤0.001). (TIF) [file pone.0099988.s004.tif]

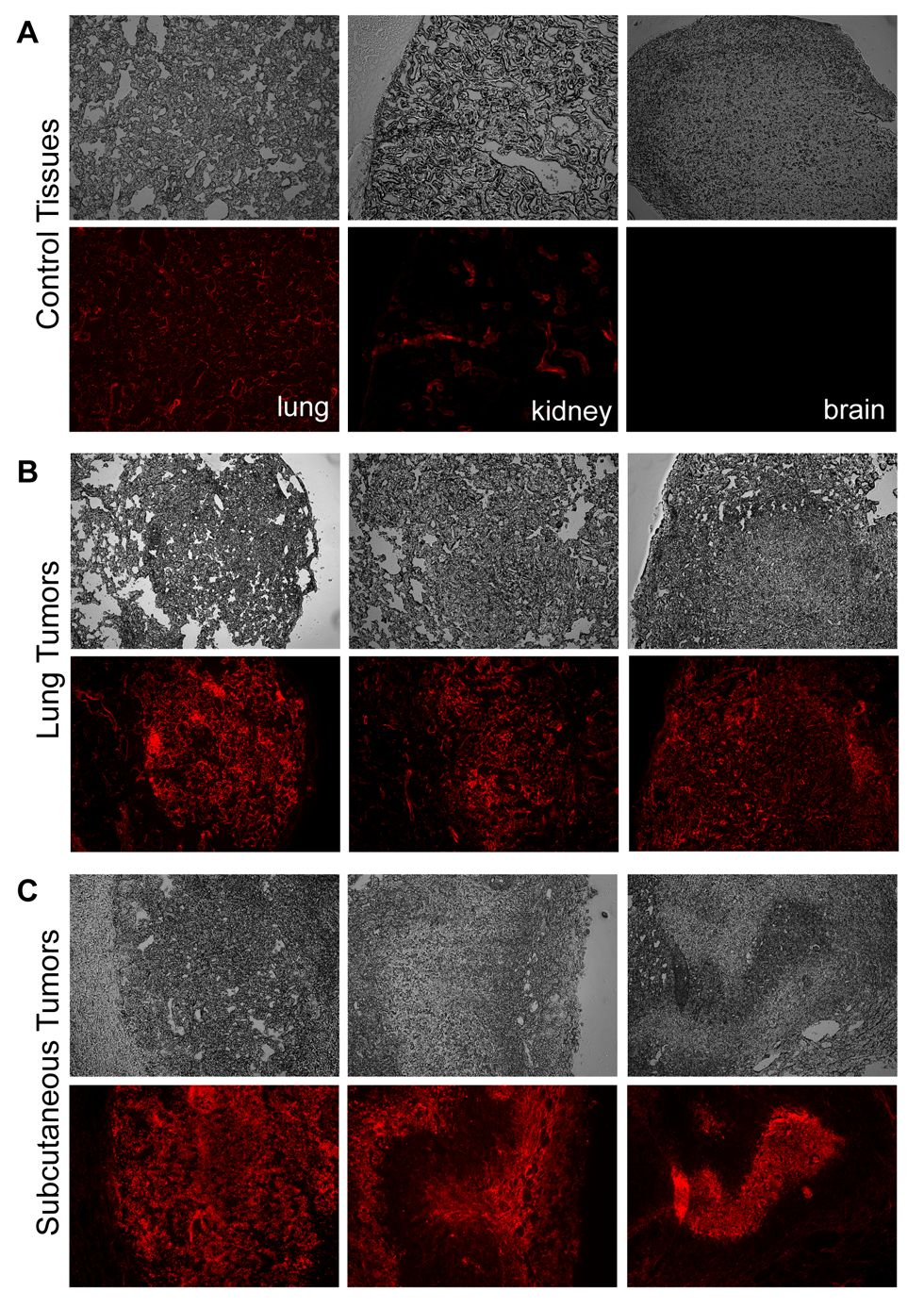

Supplement: Figure S5 — Vessel permeability of subcutaneous versus metastatic tumors. (A) Phase-contrast and corresponding fluorescence images of control tissues demonstrate Evans blue dye outlining the vessels in the lung and kidney, consistent with low levels of background dye leakage. The vessels in the brain, however, showed no leakage, consistent with the dyes inability to cross the blood brain barrier. (B) Representative images of Evans Blue dye leakage in three different lung tumors, and (C) subcutaneous tumors. Scale bar = 100 µm. (TIF) [file pone.0099988.s005.tif]

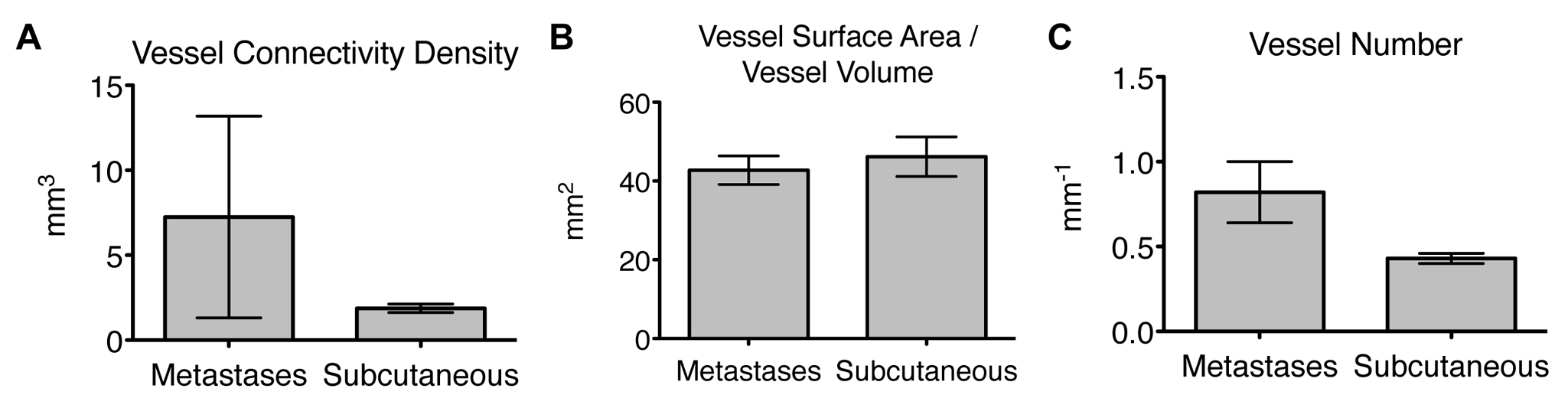

Supplement: Figure S6 — Quantification of vessel parameters of subcutaneous versus metastatic tumors. (A) There was decreased vessel connectivity in subcutaneous 344SQ-ELuc tumors compared to primary adenocarcinoma-derived p53R172HΔg/+ K-rasLA1/+ metastases as well as a decrease in vessel number (B) (not significant). Vessel surface area/vessel volume (C), however, was similar between the tumor sites indicating that the dimensions of the vessels present were comparable. Data represented as mean ± SEM. (TIF) [file pone.0099988.s006.tif]

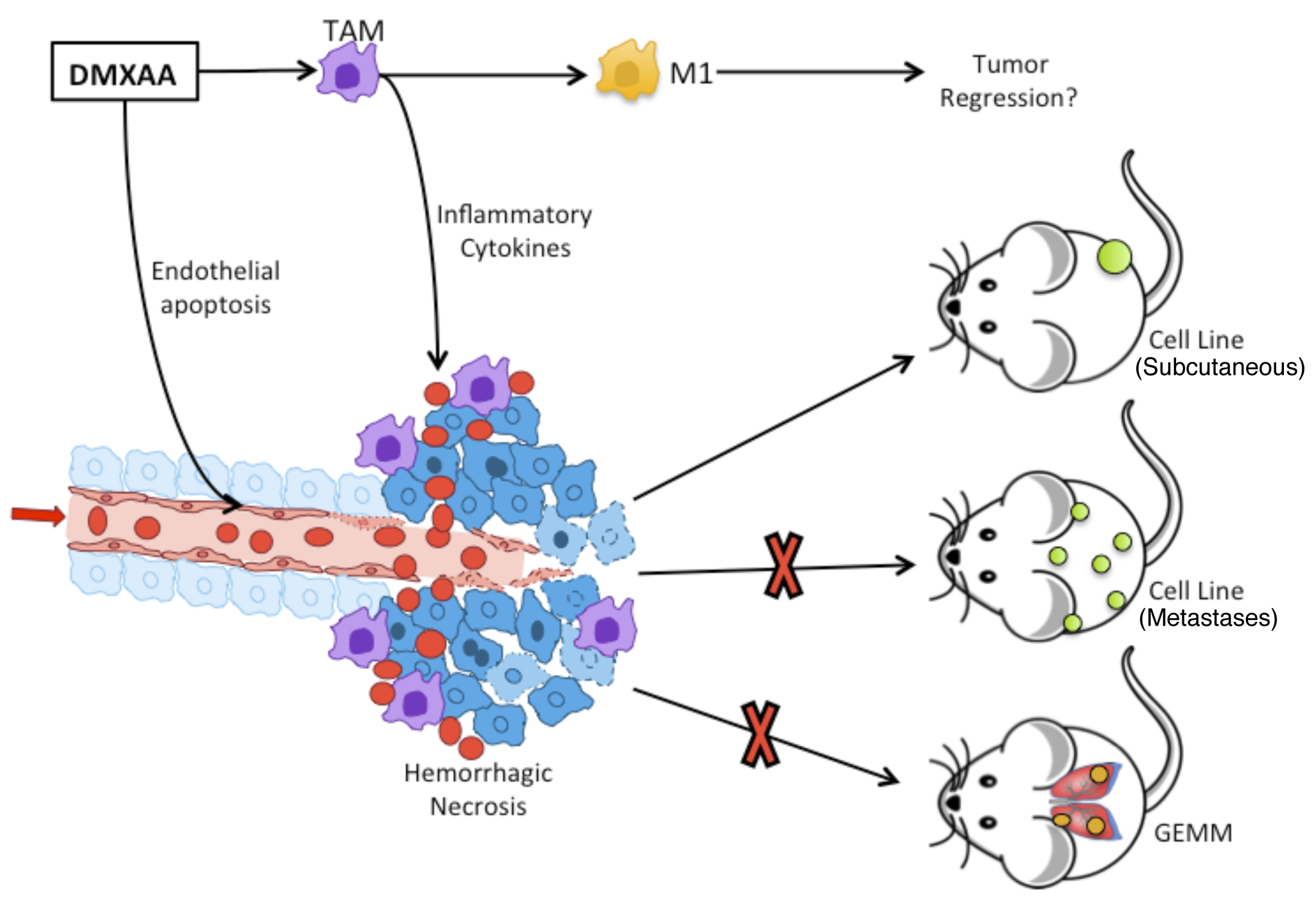

Supplement: Figure S7 — Summary of DMXAA effects in the murine NSCLC models. Induction of M2-like TAM repolarization towards an M1-like phenotype by DMXAA accompanied the rapid onset of hemorrhagic necrosis of subcutaneous tumors. In contrast, DMXAA did not exhibit vascular disrupting effects on either syngeneic metastases or spontaneously arising NSCLC in tumors in KrasLA1 GEMM model. (TIF) [file pone.0099988.s007.tif]

**
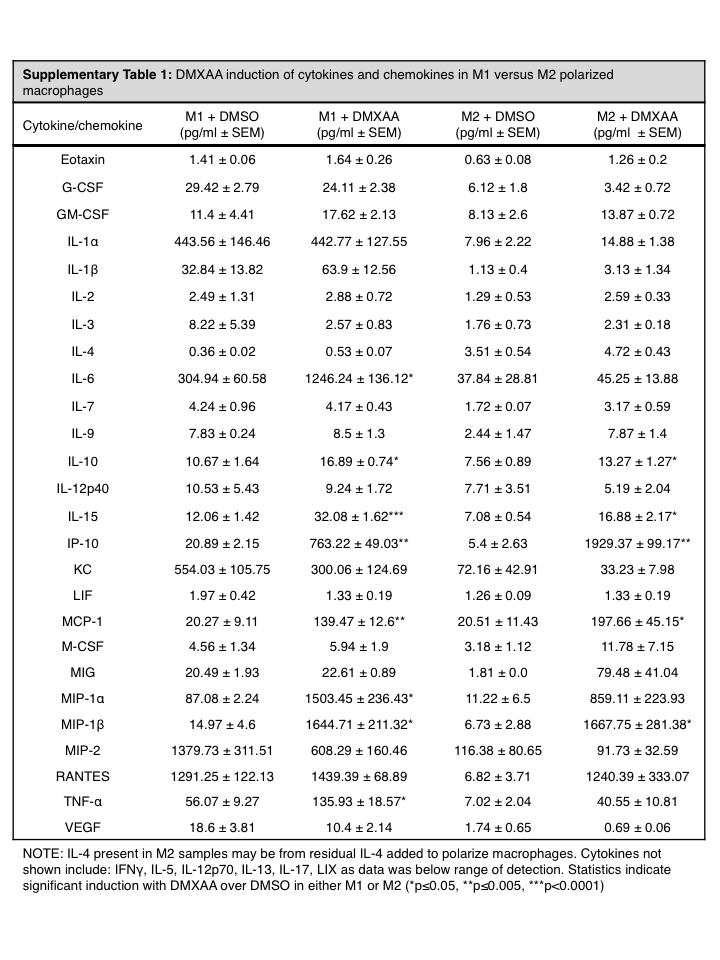
**

Supplement: Table S1 — DMXAA induction of cytokines and chemokines in M1 versus M2 polarized macrophages. Polarized BMDM (N = 3) were treated with DMXAA or DMSO control and serums were analyzed via cytokine array. Average values are shown. (DOCX) [file pone.0099988.s008.docx]
